# Anæsthetics in Veterinary Practice

**Published:** 1881-01

**Authors:** William Henry Porter

**Affiliations:** Lecturer on Surgery, Columbia Veterinary College


### ART. II.—ANÆSTHETICS IN VETERINARY PRACTICE.

The most efficient method of accomplishing anæsthesia in animals that are to be subjects of severe surgical interference, is a topic of prime importance to every one who is interested in veterinary medicine. In human practice, and with small animals, the difficulties are trivial and easily surmounted, while in horses the task has always been regarded as formidable. It is the object of this brief paper to describe a method that is simple, effectual and inexpensive. It will be remembered, that after the animal has been thrown and anæsthetics are given, it has been customary to apply the ordinary cone, made of paper and cloth, and containing a sponge or cotton soaked in sulphuric ether or chloroform ; or, in other cases, an almost air-tight nose-piece has been used. In either case both nostrils and mouth have been covered, and the expenditure of the anæsthetics has been large, but much less in the latter than in the former.

A plan, which the writer thinks may have first been used by himself, is now proposed ; it is carried out as follows: The horse having been thrown and temporarily secured, the operator immediately takes advantage of the known fact that most horses breathe through the nostrils only; he accordingly closes the inferior nostril with his hand, or that of an assistant; the animal is now, of necessity, compelled to breathe entirely through the other, or superior nostril. A small cone is now brought into use; it should be just large enough to tightly cover the superior nostril, and thus completely exclude the entrance of all air. The quantity of air required to oxidize the blood during the administration of the anæsthetic, can be governed by the inferior nostril, and yet the inhalation of the anæsthetic need not be interfered with.

When all the details of this method are precisely followed, the writer is confident that every veterinary surgeon will testify that ounces can do the work that formally required pounds. It will need some skill and practice to successfully meet all the requirements necessary to administer any anæsthetic in this way, but the same is equally true of all the methods.

As there are a few horses that can breathe through the mouth almost as well as through the nostrils, this plan of etherization will in those cases have to be aided by any apparatus that will close the mouth tightly. Some animals seem to be unable to breathe through the mouth even when completely under the influence of an anæsthetic; but the question arises, will not the majority, when completely relaxed by ether or chloroform, be able to breathe through the mouth? Although they may not be able to expire freely through the mouth, there seems to be no good reason why they cannot inpire air into the lungs through that cavity. The writer has not as yet been able to extend his observations sufficiently to positively demonstrate this point, but would be happy at any time to hear the experience of those who may have occasion to resort to an anæsthetic. The writer, by this method, was once able to bring an animal pretty well under the influence of the anæsthetic, with four drachms of sulphuric ether; just at this point the palate became relaxed and the animal breathed freely through the mouth. But by closing the mouth tightly this will be found a very economical way of administering an anæsthetic.

This simple plan will always suggest itself to those who are desirous of carrying out an ordinary operation, but are embarrassed by the expense of the anæsthetic. It is as yet an undecided question just how much can be saved.

The plan of etherizing a horse standing, or without first throwing and temporarily securing, will be a decided advance in veterinary science, but will always require a larger quantity of the anæsthetic than would be used when the animal has been thrown and placed under perfect control.

Up to the present time veterinary surgeons have devoted most of their energy to giving reasons for not using anæsthetics, which unfortunately have been principally that of the expense of the drug. A few of the reasons why anæsthetics should be universally used in severe operations are, the dangers to the life of the animal from breaking the back, rupture of the diaphragm, and shock. This last cause, shock, may at first seem a bold term.

First, the throwing of the animal can be easily accomplished, but to cast a horse and have him go through all his struggles without breaking his back, is probably a thing which no veterinary surgeon of any considerable experience has always succeeded in accomplishing. By immediately using an anæsthetic as soon as thrown, there would be the least possible chance of this accident.

The second danger is that when an animal is thrown and securely bound, the whole force of the animal's struggle seems to be exerted upon the diaphragm, it ruptures, and death instantly follows. By a speedy use of an anæsthetic this accident could not occur.

The third reason for using an anæsthetic is the condition which the writer has thought justifiable to call shock. The reason for using the term shock is the well-known fact, that horses when bound and under the knife, or when simply under the knife, "struggle and fight until they are subdued," as it is called, but this subdued condition is, without doubt, identical with the condition known as shock in human medicine. That is, the horse stands or lies trembling, as if unable to move or make further resistance; he is covered with a cold perspiration; the temperature falls, the pulse is small and fluttering, and death frequently follows these symptoms. It has often happened to the veterinary practitioner that after a horse has been cast and "peacefully submitted to the operation," when loosed from his bonds, instead of getting up as the veterinary surgeon in charge would like to have him, he has died where he lay when operated on. Death in such cases is, without doubt, from the condition termed shock. Were this accident to occur in the case of a valuable and blooded animal, the few dollars saved on the anæsthetic could never repay the loss sustained by the owner; or, the damage to the veterinary surgeon.

As a general rule it is in the high-blooded, spirited and nervous animals that this accident—death from shock—is most likely to occur.

It appears to the writer that these three fatal consequences, without mentioning any of the more trivial accidents, or even bringing up the great and all-important question of humanity to animals, would be all that is required to make the use of anæsthetics as universal in the veterinary as they have become in human medicine.

These few facts ought to lead every veterinary surgeon to make a thorough and constant study of the subject, until the use of anæsthetics can be brought to the minimum of ease, safety and expense. There is no reason why this end cannot be accomplished, and the success of comparative surgery raised to a plane, equally as high as human surgery.

WILLIAM HENRY PORTER, M. D.,
*Lecturer on Surgery, Columbia Veterinary College.*

